# Maturation of trauma systems in Europe

**DOI:** 10.1007/s00068-023-02282-0

**Published:** 2023-05-30

**Authors:** Samantha Scharringa, Suzan Dijkink, Pieta Krijnen, Inger B. Schipper

**Affiliations:** 1 Department of Surgery, Leiden University Medical Center, Leiden, The Netherlands; 2 Network Acute Care West, Leiden, The Netherlands; 3 Department of Surgery, Haaglanden Medical Center, The Hague, The Netherlands

**Keywords:** Trauma, Trauma systems, Europe, Maturation, Trauma centers, Development

## Abstract

**Purpose:**

To provide an overview of trauma system maturation in Europe.

**Methods:**

Maturation was assessed using a self-evaluation survey on prehospital care, facility-based trauma care, education/training, and quality assurance (scoring range 3–9 for each topic), and key infrastructure elements (scoring range 7–14) that was sent to 117 surgeons involved in trauma, orthopedics, and emergency surgery, from 24 European countries. Average scores per topic were summed to create a total score on a scale from 19 to 50 per country. Scores were compared between countries and between geographical regions, and correlations between scores on different sections were assessed.

**Results:**

The response rate was 95%. On the scale ranging from 19 to 50, the mean (SD, range) European trauma system maturity score was 38.5 (5.6, 28.2–48.0). Prehospital care had the highest mean score of 8.2 (0.5, 6.9–9.0); quality assurance scored the lowest 5.9 (1.7, 3.2–8.5). Facility-based trauma care was valued 6.9 (1.4, 4.1–9.0), education and training 7.0 (1.2, 5.2–9.0), and key infrastructure elements 10.3 (1.6, 7.6–13.5). All aspects of trauma care maturation were strongly correlated (*r* > 0.6) except prehospital care. End scores of Northern countries scored significantly better than Southern countries (*p* = 0.03).

**Conclusion:**

The level of development of trauma care systems in Europe varies greatly. Substantial improvements in trauma systems in several European countries are still to be made, especially regarding quality assurance and key infrastructure elements, such as implementation of a lead agency to oversee the trauma system, and funding for growth, innovation and research.

## Introduction

A trauma system is an integrated and systematic structure designed to facilitate and coordinate a multidisciplinary system response to provide an optimal care continuum for seriously injured patients [1]. In Europe, Germany was the first country to implement a trauma system in 1972, followed by many others in the late twentieth century and early twenty-first century [2]. A 2008 study [2] found substantial variation in the current stage of trauma system development and trauma surgery training among European countries, with countries tied to the Austro-German surgical tradition (Germany, Austria, Switzerland, The Netherlands, Czech Republic, Slovakia, Hungary, and Slovenia) performing best. Nine years later, in a systematic review [3] on trauma systems around the world, variation in trauma system development among European countries was still substantial.

In Europe, trauma still is the leading cause of death in people under the age of 40 [4, 5]. Hence, matured trauma systems are urgently needed to ensure high quality of trauma care. According to the World Health Organization (WHO), a mature trauma system is a system that has embedded a formal and interconnected prehospital trauma care system, has set standards for education, training, and licensing, includes appointed trauma centers that are verified and accredited by the Ministry of Health, acknowledges a lead agency to supervise trauma care, and has incorporated formal trauma care quality assurance programs [6]. Studies examining the benefits of having a mature trauma system reveal that it leads to higher survival rates [7–9], improved quality of life [10], and cost reduction for every life saved [11]. Furthermore, a mature trauma system will also benefit prevention programs, research, and education owing to the data collected in national registries [12].

This study aims to provide a current overview of trauma system maturation in European countries, based on the results of a self-assessment survey per country.

## Methods

### Study design

A survey was sent via email to 117 surgeons from 24 countries in Europe with a personal interest in trauma care development and involved in trauma, orthopedics, or emergency surgery. For the selection of participants, the country representatives of the ESTES advisory board were asked to nominated five trauma surgeons. If there was no response from the national ESTES representative, one of the authors (IBS) approached a well-known national trauma-involved surgeon of that country and asked him or her to propose five participants for their country. To limit response bias due to responses of multiple surgeons from the same hospital or region, surgeons from different hospitals in the country were approached. Primarily, trauma surgeons were invited to fill out the questionnaire. If trauma surgery was not a separate specialty or if no responses were received, orthopedic surgeons, military surgeons, general surgeons, and emergency physicians were also invited. To increase the reliability on knowledge of their own country’s trauma system, members of the European Society for Trauma and Emergency Surgery (ESTES) were first approached, followed by department directors, surgeons active in trauma research, surgeons in academic hospitals, and surgeons who were known to be active and interested in the development of their country’s trauma system. Incomplete response forms and countries from which only one single response form was received were excluded from analysis.

### Survey

Measurement of trauma system maturation was performed using a four-part self-assessment tool based on the WHO Trauma System Maturation scale, combined with a questionnaire based on the prerequisites needed for trauma system development as described by The American Association for the Surgery of Trauma (AAST), resulting in a self-developed five-part survey (Table 1). Questions in parts 1 to 4 regarding prehospital care (Part 1), facility-based trauma care (Part 2), education and training (Part 3), and quality assurance (Part 4) were designed to reflect the WHO Trauma System Maturity Index [6]. Questions in Part 5 on key infrastructure elements were designed in accordance with the Trauma System Agenda for the Future (including leadership, professional resources, education and advocacy, information management, finances, research, and disaster preparedness and response) by the AAST [13].

**Table 1:** Survey questions

Part 1: Prehospital care		
1a	What is the state of the prehospital care system in your country?	
1 pt	No formal emergency medical services	
2 pt	Formal emergency medical services available	
3 pt	Formal emergency medical services controlled by a lead agency	
1b	Is there a (dispatch) control center available to allow communication between prehospital service providers and facility-based health care providers?	
1 pt	No defined communication system	
2 pt	Coordination seen between various agencies for prehospital care delivery and hospital, but no formal system	
3 pt	Legislative mechanism in place to govern EMS and coordinate universal coverage	
1c	Is there a national emergency phone number available to reach emergency medical services?	
1 pt	Communication via phone is not available to all inhabitants throughout the country	
2 pt	Several phone numbers available throughout the country	
3 pt	One national emergency phone number available	
Part 2: Facility-based trauma care		
2a	To what extent are trauma centers installed?	
1 pt	There are no predefined criteria for hospitals regarding levels of trauma care	
2 pt	Roles of various hospitals are clearly defined regarding trauma care; dedicated trauma centers have been appointed	
3 pt	Roles are clearly defined regarding trauma care and dedicated trauma centers have been appointed; a procedure of hospital verification and accreditation is in place through the Ministry of Health and followed by the professional bodies	
2b	To what degree are human and physical resources available within a trauma care facility?	
1 pt	General human and physical trauma resources are available during office hours	
2 pt	Human and physical trauma resources are available during office hours and 24/7 in some dedicated hospitals	
3 pt	Human and physical trauma resources are available 24/7 in all hospitals	
2c	To what extent is the hospital part of a structured trauma system in your country?	
1 pt	The hospitals are working as standalones, no agreements with other prehospital organizations or clinical facilities	
2 pt	The hospitals have some agreements with other prehospital organizations or clinical facilities, no formal structure	
3 pt	Hospital communication with other trauma care providing partners is well-structured and protocolized in a formal trauma network with a leading entity (trauma center/organization)	
Part 3: Education and training		
3a	To what extent is prehospital health care personnel trained to provide trauma care?	
1 pt	No specific health care personnel trained to offer primary trauma care in the community	
2 pt	Training is not mandatory for all prehospital emergency trauma care providers, but several identified personnel are able to provide trauma care	
3 pt	Structured educational protocols such as PHTLS and mandatory training for prehospital emergency trauma care providers are implemented	
3b	To what extent is in-hospital health care personnel trained to provide trauma care?	
1 pt	No definite training requirement for clinical doctors and Emergency Department personnel	
2 pt	Some training courses available, but are not mandatory	
3 pt	Training (ATLS, ETC) is mandatory, norms for different levels of health care providers are in place	
3c	How are trauma surgeons qualified?	
1 pt	There are no dedicated trauma surgeons, visceral trauma can be done by any surgeon	
2 pt	General surgeons with experience in trauma management	
3 pt	Certified trauma surgeons: licensing and renewal is mandatory	
Part 4: Quality assurance		
4a	Is there a trauma registry implemented in your country?	
1 pt	No structural trauma registry implemented	
2 pt	Well organized local, facility-based, or regional trauma systems implemented with regular analysis	
3 pt	Nationwide or international trauma registries implemented with at least annual analysis and reports	
4b	To what degree is there a quality assurance/auditing system implemented for trauma care?	
1 pt	No formal auditing of hospitals or other care stake holders	
2 pt	Local or regional hospital or ambulance quality assurance program available; structured auditing on predetermined intervals	
3 pt	Formal quality assurance programs are in place and are mandated in prehospital and facility-based services, nationally coordinated	
4c	Protocols	
1 pt	No formal agreements or protocols for either trauma care in hospitals or ambulance services	
2 pt	Protocols for trauma care are present but may vary from hospital to hospital or region to region, no control of protocol compliance	
3 pt	Prehospital end clinical trauma protocols are the same for all hospitals and ambulance services	
Part 5: Key infrastructure elements according to the AAST Trauma System Agenda for the Future		
	For each question below, award	
	1 pt	No
	2 pt	Yes
5a	Leadership: There is a nationwide leadership council responsible for the development and improvement of the nationwide trauma system	
5b	Professional resources: There is sufficient funding for graduate medical education	
5c	Education and advocacy: There are injury awareness and prevention programs implemented in the country	
5d	Information management: The current state of the trauma system is discussed at regular regional/national meetings and in registry-based publications	
5e	Finances: There is allocated budget for trauma system development from the government	
5f	Research: Nationwide studies to improve trauma care are financially supported and coordinated by national professional scientific and/or governmental organizations	
5g	Disaster preparedness and response: Disaster preparedness protocols are readily available and trained throughout the chain of trauma care	

Parts 1 to 4 each consisted of three questions with three answer categories that were scored as 1 (lowest level of maturation) to 3 (highest level of maturation). Part 5 consisted of seven questions and was scored as 1 (absent) or 2 (present). For Parts 1–4 of the survey, the maximum attainable score was 9 per part (36 for all 4 parts together) and for Part 5 a maximum of 14. For each country, the mean score of each part is presented, as well as the end (sum) score with a minimum of 19 and a maximum of 50. Survey results per country were obtained by averaging the scores of the respondents from each country (with varying sample size). Subsequently, survey results by geographical region were obtained by averaging the scores of the countries in each region (with varying sample sizes).

### Statistical analysis

To investigate whether trauma system maturation differed by geographical region, countries were grouped into Western Europe (Austria, Belgium, France, Germany, the Netherlands, Switzerland), Northern Europe (Denmark, Finland, Ireland, Norway, Sweden, the United Kingdom), Eastern Europe (Bulgaria, Czechia, Hungary, Poland, Slovakia), and Southern Europe (Croatia, Cyprus, Greece, Italy, Portugal, Spain, Slovenia) based on the United Nations Geoscheme [14]. Differences in scores between regions were analyzed using Welch’s ANOVA together with the Games-Howell test for post hoc comparisons. To evaluate whether scores on different parts were correlated, a correlation matrix was made based on Pearson’s correlation coefficient. An association was considered absent for correlation coefficients < 0.2, weak between 0.2–0.4, moderate between 0.4–0.6, strong between 0.6–0.8 and very strong if > 0.8. Statistical significance was set at *p* < 0.05. All analyses were performed using R software (version 4.2.1).

## Results

**Fig. 1:**
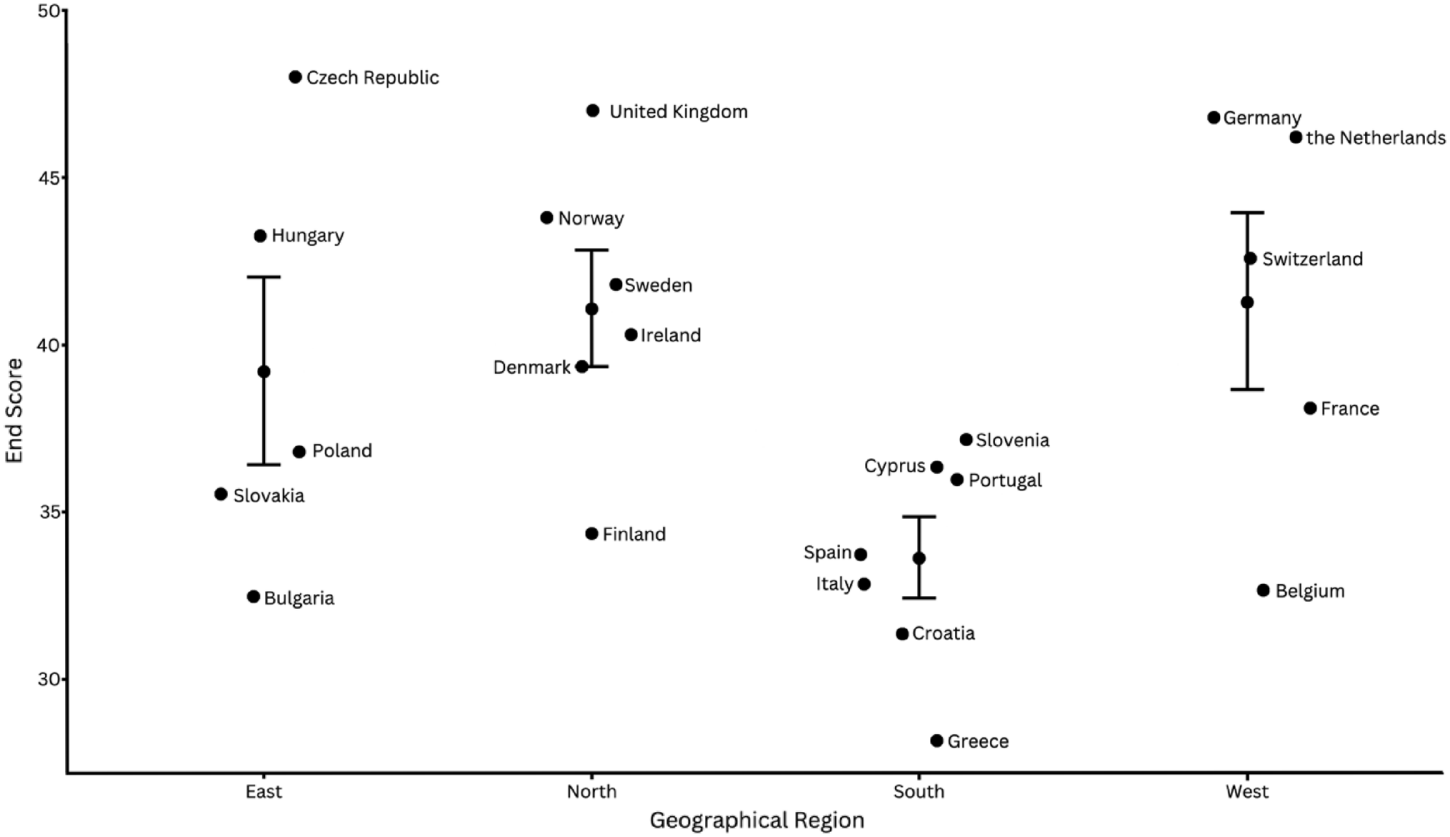
Survey end scores per country. The bars represent the mean end scores ± standard deviation of the countries within each geographical region

**Fig. 2:**
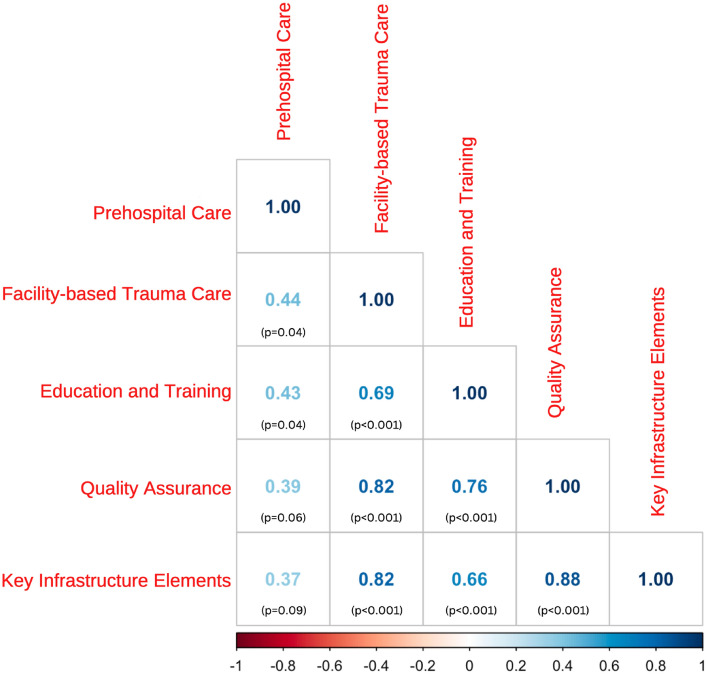
Pearson’s correlation coefficient matrix for the separate parts of the questionnaire

111 complete responses were received from 23 countries (94.9% response rate). One country did not respond and was subsequently not included in the analysis. Responses from the United Kingdom only represented Scotland. The responses per country are listed in Table 2. End scores ranged between 28.2 and 48.0, with mean of 38.5 and standard deviation (SD) of 5.6. Czechia, the United Kingdom, and Germany had the highest end scores, whereas Bulgaria, Croatia, and Greece had the lowest. Welch’s ANOVA showed statistically significant overall difference between geographical regions (*p* = 0.03). The Games-Howell post hoc test showed statistically significant end scores between the Northern and Southern regions (*p* = 0.03), but not between other regions (Fig. 1). All aspects of trauma care maturation were strongly correlated (*r* > 0.6) except prehospital care (*r* = 0.37) (Fig. 2).

**Table 2:** Survey results by country, per survey section

Country	No. of respondents	Prehospital care	Facility-based trauma care	Education and training	Quality assurance	Key infrastructure elements	End score
Czechia	2	9.0	8.5	9.0	8.5	13.0	48.0
The United Kingdom	2	8.5	9.0	7.5	8.5	13.5	47.0
Germany	4	8.5	9.0	9.0	8.5	11.8	46.8
The Netherlands	5	8.8	8.2	9.0	7.8	12.4	46.2
Norway	5	8.8	8.8	7.4	7.2	11.6	43.8
Hungary	3	8.3	8.0	8.7	6.7	11.7	43.3
Switzerland	5	8.0	7.4	8.2	7.4	11.6	42.6
Sweden	4	8.5	7.0	8.0	7.8	10.5	41.8
Ireland	3	8.7	6.3	6.7	7.3	11.3	40.3
Denmark	3	8.0	7.7	6.7	6.0	11.0	39.3
France	9	8.1	7.7	5.7	6.2	10.4	38.1
Slovenia	5	8.2	6.6	8.0	5.0	9.4	37.2
Poland	5	8.2	7.8	6.8	4.4	9.6	36.8
Cyprus	4	7.8	6.0	8.0	5.8	8.8	36.3
Portugal	5	9.0	6.6	6.4	4.6	9.4	36.0
Slovakia	4	8.3	6.0	6.3	4.5	10.5	35.5
Finland	5	7.8	6.2	5.4	5.0	10.0	34.4
Spain	10	7.8	6.1	5.7	5.3	8.8	33.7
Italy	5	8.8	6.0	6.2	4.2	7.6	32.8
Belgium	9	7.9	5.3	5.9	4.6	9.0	32.7
Bulgaria	2	7.5	6.0	6.0	4.5	8.5	32.5
Croatia	3	9.0	4.7	6.0	3.3	8.3	31.3
Greece	9	6.9	4.1	5.2	3.2	8.8	28.2
Total mean ± SD (range)		8.2 ± 0.5 (6.9–9.0)	6.9 ± 1.4 (4.1–9.0)	7.1 ± 1.2 (5.2–9.0)	5.9 ± 1.7 (3.2–8.5)	10.3 ± 1.6 (7.6–13.5)	38.5 ± 5.6 (28.2–48.0)

### Part 1: Prehospital care

Czechia, Portugal, and Croatia scored the maximum score of 9, whereas Greece had the lowest score of 6.9 (Table 2). Regarding the prehospital system (Q1a), 21.7% countries agreed that their formal EMS is controlled by a lead agency (3 points). Other countries varied in responses between 2 and 3 points, and Greece was the only country where multiple respondents gave 1 point (no formal EMS). Concerning dispatch control center availability (Q1b), 39.1% agreed that their country has a legislative mechanism in place to govern EMS and coordinate universal coverage (3 points). 82.6% of countries have one national emergency phone number available (Q1c); the respondents of France, Greece, Ireland, and Spain gave non-unanimous answers to this question.

### Part 2: Facility-based trauma care

Germany and the United Kingdom had the highest score, whereas Greece scored the lowest (Table 2). With regard to the installation of trauma centers (Q2a), 5 out of 23 countries (21.7%) have dedicated trauma centers that are verified and accredited by the Ministry of Health (3 points), 2 out of 23 (8.7%) have trauma centers that are not verified and accredited by the Ministry of Health (2 points), and 1 country has no predefined criteria for hospitals regarding levels of trauma care (1 point). Respondents from other countries disagreed on the answers. On the topic of availability of resources (Q2b), 17.4% of countries have 24/7 human and physical resources available (3 points) and 17.4% have 24/7 availability in several dedicated hospitals (2 points). As to the presence of a formal trauma system structure (Q3c), 5 out of 23 countries (21.7%) have a formal trauma care network led by an organization (3 points) and 3 out of 23 countries (13%) do not have a formal network but hospitals do have agreements with other prehospital organizations or clinical facilities (2 points).

### Part 3: Education and training

Czechia, Germany, and the Netherlands scored 9 points for education and training, whereas Greece had the lowest score of 5.2 (Table 2). With respect to prehospital personnel training (Q3a), 39.1% of countries have mandatory training and structured educational protocols implemented for prehospital emergency trauma care providers (3 points). As to the extent of trauma care training (i.e., ATLS, ETC) (Q3b), 30.4% stated that training is mandatory (3 points) and 13% stated that courses are available but not mandatory (2 points). Discrepancies among responses were observed for France, Greece, and Spain, where the answers varied from 1 point (no definite training requirement for clinical doctors and Emergency Department personnel) to 3 points (training is mandatory). Concerning trauma surgeon qualification (Q3c), 13% of countries responded that trauma surgeons are certified with mandatory licensing and renewal, 26% responded that trauma surgeons are general surgeons with experience in trauma management, and 8.7% responded that there are no dedicated trauma surgeons.

### Part 4: Quality assurance

No country scored the maximum score of 9. The highest score was 8.5 for Czechia, the United Kingdom and Germany. By the respondents of 30.4% of the countries, it was disclosed that they have a national trauma registry (3 points), 8.7% have regional registries (2 points), and 21.7% do not have registries (1 point). Maximal variation in answers was observed for Q4b: the implementation of quality assurance/auditing systems. Among the countries from which all respondents gave the same score, the respondents from Czechia and the United Kingdom agreed that formal programs are in place and are nationally coordinated (3 points) and Bulgaria and Denmark agreed that there is structured auditing at predetermined intervals (2 points). In terms of protocols, 78.3% of the countries gave non-unanimous answers. The respondents from 21.7% of countries gave corresponding answers, all claiming that their countries have protocols for trauma care but that these may vary between hospitals and/or regions (2 points).

### Part 5: Key infrastructure elements according to the AAST

No country scored the maximum score of 14; the United Kingdom had the highest score (13.5) on this section. 39.1% of countries scored ≥ 11, and 60.9% of countries scored between 7 and 10.9. Italy scored lowest with 7.6 (Table 2). Although the answers on the topic were varying within most countries, the majority of respondents reported that there is no nationwide leadership council responsible for the development and improvement of the nationwide trauma system (Q7a), that there are injury awareness and prevention programs implemented in the country (Q7c), that there is no governmental budget allocation for trauma system development (Q7e), that studies to improve trauma care are not financially supported or coordinated by professional organizations (Q7f), and that disaster preparedness protocols are readily available and trained throughout the chain of trauma care (Q7g).

## Discussion

A well-functioning and well-developed trauma system is critical for improving patient survival and outcome. The results of this self-assessment survey show that the overall trauma system maturity score for the European countries varied between 28.2 and 48.0 points on a scale from 19 to 50 points. This reflects that the variation in the level of development of trauma care systems in Europe is substantial, with countries in Northern Europe evaluating their trauma system development significantly higher than countries in Southern Europe, suggesting a geographical gap in the degree of trauma system maturity. Furthermore, variation in maturity between elements of trauma care was also observed within countries, with most countries evaluating their prehospital care as well developed and their quality assurance as least developed.

The need to understand and value trauma systems has been long called for. Trauma remains one of the leading causes of death worldwide, and the most common cause of death in Europeans younger than 40 [5]. Lessons from the World Wars [15, 16], the polio epidemic [17], and the coronavirus pandemic [18] have stressed the value of a well-developed and well-organized healthcare system, including the presence of a mature trauma system to reduce mortality. Yet, as stated by the International Orthopaedic Trauma Association in 2019, “while the interest in developing trauma care is growing, the overall adoption is low” [19].

Disparities in the degree of trauma system maturation in Europe have previously been demonstrated [2, 3, 20, 21]. A previous self-assessment in 2008 showed that central European countries with ties to the Austro-German surgical tradition (Germany, Austria, Switzerland, the Netherlands, Czechia, Slovakia, Hungary, and Slovenia) rated their trauma systems as advanced in terms of trauma system development and trauma surgery specialization [2]. However, by 2017, this difference was no longer significant, as other countries have also improved in the domains of trauma surgery specialization and overall trauma system development [3]. It has been proposed that the pace at which trauma systems in a country develop and are organized is largely determined by the occurrence of national disasters, or by its most pressing national healthcare challenge [17]. For example, changes to the trauma system in the Netherlands were initiated after a devastating plane crash that highlighted the lack of organization between prehospital and in-hospital care, while improvement of the Spanish trauma system was motivated by the increase in road traffic accidents [21].

Each country faces its own challenges with regard to trauma system implementation. While some trauma systems must provide trauma care to highly densely populated areas, others rely on helicopter transport to cope with long distances and environmental inaccessibility [20]. Aside from geographical region variations, trauma system maturity and design may also differ substantially between similar geographical regions. One example concerns the Netherlands and Belgium. In agreement with a 2003 study [22], Belgian surgeons evaluated their trauma system as being less mature than that in the Netherlands. Although advancements have been made since 2003 [3], surgeons still report that there are no set criteria for trauma care levels, that trauma surgery is not a specialization, and that quality assurance is not implemented. Another example is differences in trauma system maturity between the Nordic countries. Although trauma surgery is not recognized as an independent specialization in any of the Nordic countries, variation in trauma systems has been observed among the individual countries. Consistent with previous findings [23, 24], Norwegian trauma systems have the highest level of trauma system maturity, owing to the availability of funding programs for research and the implementation of a trauma team training program. While both Finland and Sweden lack a lead agency to oversee the trauma system and research funding, evidence suggests that trauma care is less developed in Finland, as only 20% of trauma-receiving hospitals have trauma teams [20].

Aside from regional and geographical challenges, several generic challenges can be distinguished with regard to the development of trauma systems in Europe. First, despite recommendations and guidelines, the enforcement of trauma education and training is valued suboptimal by most participating countries. While proper education and training are also paramount for a well-functioning trauma system, our survey results suggest that trauma education is not considered equally important as prehospital and in-hospital care. Perpetuating factors in this issue include lack of funding, lack of resources, lack of interest [20], and the absence of quality control audits [25]. Second, the absence of a lead agency and quality assurance programs hinders progression. According to the Trauma Systems Agenda for the Future, the fragmentation of trauma leadership is a major impediment to the development of a national trauma system [13]. An advantage of having a clear lead agency is that it can advise the government on the development of their trauma system and to provide support. The lack of a lead agency would be challenging to maintain a national overview and would have consequences for funding and research. Third, the need to define and appoint trauma centers is still unfulfilled in several countries. Politics and economics aside, a contributing factor to this matter is the lack of recognition of trauma surgery as a separate specialization. Although the need for a trauma surgery subspecialty might seem trivial for countries that lack funding, facilities, human resources or a mature trauma system, it has been demonstrated that having dedicated trauma surgeons benefit patient safety and quality of care [26]. Additionally, dedicated trauma surgeons may also serve as ambassadors of public safety by raising awareness through research and prevention programs [27, 28]. Regarding the appointment of dedicated trauma centers, evidence suggests that severely injured patients—specifically those with head injury, thorax injury, or signs of shock—benefit from direct transport to a Level 1 trauma center [29–31]. Keeping in mind that trauma causes a high burden of death and disability, it is strongly recommended for countries to strive to implement a classification system for trauma care levels.

### Limitations

Due to the study design, a substantial response bias cannot be ruled out. The scores presented in this study reflect a subjective evaluation, and the experience and knowledge of selected surgeons with regard to their country’s trauma system. Therefore, the accuracy of the presented trauma systems evaluation cannot be guaranteed, and the results for individual countries should be interpreted with caution. Additionally, there is sampling bias. We tried to minimize this bias by approaching surgeons from different hospitals. However, due to the anonymous nature of the survey, this cannot be guaranteed. Furthermore, considering that the survey was sent electronically via email, it cannot be ruled out that other people than those intended filled out the survey. Moreover, the number of respondents differed between countries, ranging from two to ten, which could have led to an under- or over-estimation of end scores as lower numbers of respondents, more reflect subjective insights. Lastly, the survey email itself received criticism as it caused confusion surrounding the definition of “trauma surgeon,” as trauma surgery is not always regarded as a separate specialty in selected countries. It is unclear whether this played a role in limiting the number of participating countries or influenced the results in any way. Nevertheless, this survey is the first since a long time to provide an, albeit subjective, impression of the maturation of trauma systems throughout Europe, and as such provides a basis for further improvement and future research on quality of trauma system care.

## Conclusion

The ways in which trauma systems have developed in Europe vary significantly. Results show that representatives of most countries rate their trauma system as substantially matured for one or more key determinants, reflecting the importance attached to development of high-quality trauma systems. Yet this study also indicates that still improvements are to be made. Most countries have well-organized prehospital care, but trauma systems in some countries need improvements in other elements such as facility-based trauma care, education and training, and quality assurance. Regarding key infrastructure elements, multiple countries reported a lack of a lead agency to oversee the trauma system, insufficient funding for growth, and inadequate trauma research funding and coordination. Keeping in mind that trauma has a high burden of death and disability, and that evidence shows that having a well-developed trauma system is effective in reducing mortality and improving patient outcomes, it is imperative that countries strive to implement a trauma system that includes specialized training and quality assurance programs.

## Data Availability

Due to the sensitivity of the results and their potential political significance, data are not openly available.

## Appendix 1: Survey answers per respondent per subquestion

See Table 3.

**Table 3:** Answers per respondent per subquestion

Country	Part 1			Part 2			Part 3			Part 4			Part 5						
	1a	1b	1c	2a	2b	2c	3a	3b	3c	4a	4b	4c	5a	5b	5c	5d	5e	5f	5g
Belgium	2	2	3	1	3	2	2	2	2	1	1	2	1	2	1	1	1	1	1
	3	3	3	1	2	2	2	2	1	1	1	2	1	1	2	1	1	1	2
	3	2	3	1	2	2	2	2	2	1	1	2	2	2	1	1	1	1	2
	3	3	3	2	3	0	2	2	2	1	1	2	0	0	0	0	0	0	0
	3	3	3	1	3	2	2	2	3	2	2	2	1	2	1	2	1	1	1
	2	2	3	1	2	2	2	2	1	3	1	2	2	2	2	2	1	2	2
	2	3	3	1	2	2	2	2	1	1	1	1	1	1	1	2	1	1	1
	2	2	3	1	2	2	2	3	1	1	1	2	1	2	2	2	1	1	2
	2	2	3	2	2	2	3	3	1	2	2	2	2	1	2	2	2	2	2
Bulgaria	3	2	3	2	2	2	2	2	2	1	2	1	1	2	2	1	1	1	2
	2	2	3	2	2	2	2	2	2	1	2	2	1	1	1	1	1	1	1
Croatia	3	3	3	1	2	2	3	2	1	1	1	2	1	1	1	1	1	1	1
	3	3	3	1	2	1	2	2	2	1	1	1	1	2	2	1	1	1	1
	3	3	3	1	3	1	2	2	2	1	1	1	1	2	2	1	1	1	1
Cyprus	2	3	3	2	2	2	3	3	2	2	2	2	1	1	1	1	1	1	1
	2	1	3	1	1	1	3	3	2	2	1	1	1	1	1	1	1	1	1
	3	3	3	2	3	3	3	3	2	2	3	2	1	2	2	1	1	1	2
	2	3	3	2	2	3	3	3	2	2	2	2	2	2	1	1	2	1	2
Czech Republic	3	3	3	3	3	3	3	3	3	3	3	3	2	2	2	2	2	2	2
	3	3	3	3	2	3	3	3	3	3	3	2	2	2	2	2	1	1	2
Denmark	2	3	3	2	2	2	3	2	2	1	2	2	1	1	2	2	1	1	2
	2	2	3	3	2	3	3	3	2	3	2	2	2	1	2	2	1	1	2
	3	3	3	3	3	3	1	2	2	2	2	2	2	2	2	1	1	2	2
Finland	2	3	3	3	2	3	3	2	1	2	2	2	1	2	2	2	1	1	2
	2	2	3	1	2	2	2	2	1	2	1	2	1	1	2	1	1	1	2
	3	3	3	2	2	3	3	2	1	1	1	2	1	2	2	1	1	1	2
	2	3	3	2	2	2	2	2	1	2	1	2	1	2	1	2	1	1	2
	2	2	3	1	2	2	2	2	1	2	1	2	1	1	2	2	1	1	2
France	3	2	3	2	2	3	2	2	1	2	2	2	1	2	2	2	1	1	2
	3	2	3	2	2	2	2	2	1	2	1	2	1	1	2	1	2	2	2
	3	3	2	3	3	3	2	2	1	1	3	2	1	1	2	2	1	1	2
	3	3	3	3	2	3	2	2	2	3	1	2	1	1	2	2	0	2	2
	3	3	3	3	2	3	3	3	2	3	3	2	1	1	2	2	2	2	2
	3	3	3	2	2	3	2	2	2	1	2	3	1	1	1	1	1	1	2
	2	3	2	2	2	2	2	1	1	2	1	2	1	1	1	1	1	1	2
	0	3	3	3	3	3	3	2	2	2	2	3	2	2	2	2	2	2	2
	3	3	3	3	3	3	2	2	1	2	3	2	0	2	2	2	0	2	2
Germany	3	3	3	3	3	3	3	3	3	3	3	3	2	1	1	2	1	2	2
	3	3	3	3	3	3	3	3	3	3	3	3	2	1	2	2	2	2	2
	2	3	3	3	3	3	3	3	3	3	2	2	1	2	1	2	1	1	2
	2	3	3	3	3	3	3	3	3	3	3	3	2	1	2	2	2	2	2
Greece	1	3	3	1	2	1	2	2	1	1	1	1	2	1	2	1	1	1	1
	2	2	3	1	2	2	3	2	1	1	1	1	2	1	1	1	1	1	1
	2	2	3	1	2	1	3	1	1	1	1	1	2	1	1	1	1	1	2
	1	3	3	1	1	2	2	2	1	1	1	1	2	1	2	2	1	1	2
	3	2	3	2	1	2	2	2	3	1	1	3	1	1	1	1	1	1	1
	2	2	3	1	2	1	2	1	1	1	1	1	1	1	1	1	1	1	2
	1	1	2	2	2	1	2	2	2	1	2	1	1	2	2	2	1	1	1
	2	3	3	1	0	0	3	2	1	0	1	1	2	1	1	1	1	1	1
	1	3	3	1	2	2	1	1	1	1	1	1	2	2	1	1	1	1	1
Hungary	3	3	3	3	3	3	3	3	3	2	2	3	2	2	2	2	2	2	2
	3	3	3	3	2	3	3	3	3	2	3	2	2	2	2	1	1	1	1
	2	2	3	2	3	2	3	3	2	2	2	2	2	2	2	2	1	1	1
Ireland	3	3	3	2	2	2	3	3	1	3	2	2	2	1	2	2	2	2	1
	3	3	3	2	2	3	3	2	1	3	2	2	2	1	1	2	2	1	1
	3	3	2	2	2	2	3	3	1	3	3	2	2	1	2	2	2	2	1
Italy	3	3	3	3	2	0	2	2	2	1	2	2	2	1	1	2	2	1	1
	3	3	3	2	2	3	3	2	1	1	1	2	1	1	2	1	1	1	1
	2	3	3	0	0	3	3	3	2	0	0	2	0	0	0	0	1	2	1
	3	3	3	2	2	3	3	2	1	1	1	2	1	1	1	1	1	1	2
	3	3	3	3	2	3	2	2	1	2	2	2	1	1	2	1	1	1	1
Netherlands	3	3	3	3	2	3	3	3	3	3	3	3	2	2	1	2	1	1	2
	3	3	3	3	3	3	3	3	3	3	3	2	2	2	2	2	2	1	2
	3	3	3	2	2	3	3	3	3	3	2	2	1	1	2	2	1	1	2
	3	2	3	3	2	3	3	3	3	3	3	2	2	2	2	2	2	2	2
	3	3	3	3	3	3	3	3	3	3	2	2	2	2	2	2	2	2	2
Norway	2	3	3	3	3	3	3	3	2	3	3	3	2	2	2	2	2	2	2
	3	3	3	3	3	3	2	3	2	3	3	2	2	2	2	2	2	1	1
	3	3	3	3	3	3	2	3	2	3	2	2	2	1	2	2	1	1	2
	3	3	3	3	3	0	2	3	2	3	2	2	2	2	1	2	1	2	1
	3	3	3	3	3	3	3	3	2	3	2	2	2	1	2	0	1	2	2
Poland	2	1	3	3	2	3	2	2	1	1	2	2	1	1	2	1	1	1	1
	3	3	3	3	2	2	3	2	2	1	1	2	2	2	1	1	2	1	1
	3	3	3	3	2	2	2	2	3	1	2	2	1	2	2	1	1	1	2
	2	3	3	3	3	3	2	2	2	1	1	1	2	1	2	2	2	2	1
	3	3	3	3	2	3	3	3	3	1	2	2	1	1	1	1	1	1	2
Portugal	3	3	3	2	2	2	3	2	2	1	1	2	2	1	2	1	2	1	1
	3	3	3	3	3	3	3	3	1	1	3	3	2	1	2	2	1	1	2
	3	3	3	2	2	2	2	2	1	1	1	2	2	1	2	1	1	1	1
	3	3	3	2	2	2	3	2	1	1	1	2	1	1	1	2	1	1	1
	3	3	3	2	2	2	3	2	2	1	1	2	2	1	2	1	1	1	1
The United Kingdom	2	3	3	3	3	3	3	3	2	3	3	2	2	2	1	2	2	2	2
	3	3	3	3	3	3	3	2	2	3	3	3	2	2	2	2	2	2	2
Slovakia	2	3	3	2	2	2	1	2	1	1	1	2	2	1	2	1	1	1	1
	3	3	3	2	2	2	2	2	3	1	1	1	1	1	2	2	1	1	2
	2	2	3	2	3	2	2	2	3	2	3	3	2	2	2	2	1	1	2
	3	3	3	1	2	2	1	3	3	1	1	1	2	1	2	2	1	1	2
Slovenia	3	3	3	2	3	3	3	3	3	2	2	2	1	1	1	2	1	1	2
	2	3	3	2	2	3	3	3	2	1	1	2	1	2	2	1	1	1	2
	2	3	3	2	2	2	2	2	2	1	2	2	1	1	2	1	1	1	2
	2	3	3	2	2	2	3	3	3	1	1	3	1	1	2	1	1	1	2
	2	3	3	1	3	2	3	2	3	1	2	2	1	2	2	1	1	1	2
Spain	3	2	3	2	2	2	3	3	2	2	1	2	1	1	1	1	1	1	2
	3	3	3	3	3	3	3	2	2	2	2	2	1	1	2	2	1	2	2
	3	3	2	1	2	2	3	2	1	1	1	2	1	1	2	1	1	1	1
	3	2	2	2	2	2	3	2	2	2	2	2	2	1	1	2	1	1	2
	2	2	2	1	2	2	2	2	1	3	1	2	2	1	1	2	1	1	1
	2	2	2	1	3	2	1	1	1	2	1	2	2	1	2	1	1	1	1
	3	3	3	1	3	2	2	2	1	1	1	2	1	1	1	1	1	1	1
	2	3	3	2	2	3	2	2	1	2	2	3	1	1	2	1	1	1	2
	3	2	3	2	1	2	2	2	2	1	2	2	1	1	2	1	1	1	2
	3	3	3	2	2	3	2	2	1	1	2	2	1	1	2	1	1	1	1
Sweden	3	3	3	2	3	3	3	3	2	3	3	2	1	2	1	2	1	1	2
	3	3	3	2	3	2	3	3	2	3	2	2	1	2	2	2	1	1	2
	3	3	3	1	3	3	3	3	2	3	3	3	1	1	2	2	2	1	2
	2	2	3	1	3	2	3	3	2	3	2	2	1	2	2	2	1	1	1
Switzerland	2	2	3	3	3	2	3	3	3	3	2	2	2	1	2	2	2	1	2
	2	3	3	2	3	2	3	3	3	3	3	2	2	2	2	2	1	1	2
	2	3	3	3	2	2	3	3	2	3	3	2	2	2	2	2	1	1	2
	3	3	3	2	2	2	3	2	1	3	3	2	2	1	2	2	1	2	1
	3	2	3	3	3	3	3	3	3	2	2	2	1	2	2	2	1	1	2
